# Potential Effects on Cardiorespiratory and Metabolic Status After a Concurrent Strength and Endurance Training Program in Diabetes Patients — a Randomized Controlled Trial

**DOI:** 10.1186/s40798-016-0052-1

**Published:** 2016-08-11

**Authors:** Daniela Bassi, Renata Gonçalves Mendes, Vivian Maria Arakelian, Flávia Cristina Rossi Caruso, Ramona Cabiddu, José Carlos Bonjorno Júnior, Ross Arena, Audrey Borghi-Silva

**Affiliations:** 1 Cardiopulmonary Physiotherapy Laboratory, Physical Therapy Department, Federal University of Sao Carlos, Rodovia Washington Luis Km 235, Sao Carlos, SP Brazil; 2 Department of Interunits of Bioengineer, University of São Paulo, São Paulo, SP Brazil; 3 Department of Medicine, Federal University of São Carlos, São Paulo, SP Brazil; 4 Integrative Physiology Laboratory, College of Applied Health Sciences, University of Illinois Chicago, Chicago, IL USA; 5 Department of Physical Therapy, College of Applied Health Sciences, University of Illinois Chicago, Chicago, IL USA

**Keywords:** Diabetes mellitus, Concurrent training program, HbA1c, Oxygen uptake, Metabolic profile

## Abstract

**Background:**

Concurrent aerobic and resistance training (CART) programs have been widely recommended as an important strategy to improve physiologic and functional performance in patients with chronic diseases. However, the impact of a personalized CART program in patients with type 2 diabetes (T2D) requires investigation. Therefore, the primary aim of the current study is to investigate the impact of CART programs on metabolic profile, glycemic control, and exercise capacity in patients with diabetes.

**Methods:**

We evaluated 41 subjects with T2D (15 females and 19 males, 50.8 ± 7 years); subjects were randomized into two groups; sedentary (SG) and CART (CART-G). CART was performed over 1.10-h sessions (30-min aerobic and 30-min resistance exercises) three times/week for 12 weeks. Body composition, biochemical analyses, peripheral muscular strength, and cardiopulmonary exercise testing were primary measurements.

**Results:**

The glycated hemoglobin HbA1c (65.4 ± 17.9 to 55.9 ± 12.7 mmol/mol), cholesterol (198.38.1 ± 50.3 to 186.8 ± 35.1 mg/dl), and homeostasis model assessment insulin resistance (HOMA-IR) (6.4 ± 6.8 to 5.0 ± 1.4) decreased in the CART-G compared to the SG. Although body weight did not significantly change after training, skinfold measurement indicated decreased body fat in the CART-G only. CART significantly enhanced muscle strength compared to the SG (*p* < 0.05). CART was also associated with significant increase in peak oxygen uptake and maximal workload compared to the SG (*p* < 0.05).

**Conclusions:**

These data support CART as an important strategy in the treatment of patients with T2D, producing both physiologic and functional improvements.

**Trial Registration:**

Ensaiosclinicos.gov.br, RBR492q8z

## Key Points

- Concurrent aerobic and resistance training has important translational implications for the management of diabetic patients
- A 12-week supervised, individualized, and personalized concurrent aerobic and resistance training improves glycemic control, body composition, and functional capacity parameters, including oxygen uptake and muscle strength, in diabetic patients
- Changes in peak VO_2_ could predict 21 % of the changes expected for HbA1c, which can explain the link between metabolic profile and exercise performance using a concurrent aerobic and resistance training program

## Background

In Brazil, type 2 diabetes (T2D) currently assumes a prominent position among the non-communicable diseases, affecting 11.3 million people in 2011; projections indicate that this number is likely to triple by 2030 [1]. In view of this scenario, lifestyle interventions that include change in diet, exercise, and education hold significant benefit in reducing cardiovascular risk factors in patients with T2D [2].

In particular, exercise training (ET) has long been considered a cornerstone in the management of T2D [3]. Aerobic and resistance activities produce differing metabolic and molecular responses [4]. It is well established that the exercise increases skeletal muscle glucose uptake through an insulin-independent pathway [5], indicating muscle contraction directly impacts glucose homeostasis. In fact, skeletal muscle plays a primary role in glycemic control and metabolic homeostasis, being a predominant site of glucose disposal under insulin-stimulated conditions [6]. Although aerobic and resistance exercise are individually capable of promoting substantial health benefits, the signaling pathways and physiologic effects are specific to training mode [4]. Aerobic exercise improves mitochondrial biogenesis [7], the intrinsic oxidative capacity of muscle, and the delivery and utilization of substrates in working muscle [8, 9]. Conversely, resistance exercise induces morphological and neurological adaptations which contribute to changes in muscle neural firing pattern, size (i.e., hypertrophy), and function (i.e., strength and power) [10, 11].

Ideally, both aerobic and resistance components are included in an individualized ET program [3]. Inclusion of both aerobic and resistance ET improves aerobic capacity/performance, muscle strength, and endurance and preserves muscle mass [12], all importantly contributing to improvements in cardiovascular risk profile, morbidity, and mortality [13].

In a well-designed study with a statistically robust sample size, Sigal et al. observed that combined aerobic and resistance training over a long period was superior to either mode in isolation in improving glycemic control [14]. However, in this study, although there was a progression of training intensity, combined training was only individually applied during the first 4 weeks, and the type, order, and progression of resistance exercises prescribed were not clear and were not well described. Church et al. observed that a combined exercise training program was more effective in improving HbA1c [15]. Although the authors clearly describe the intensity of aerobic and resistance training as well as the types of resistance exercises, the daily sequence of training as well as progression was unclear. Such details are very important when considering the optimization of results, especially for short-duration training programs.

In 1980, Hickson was the first to investigate the effects of a concurrent exercise training program, which combined aerobic and resistance components during the same session [16]. Recently, programs that combine aerobic and resistance training have been investigated with a focus on optimizing outcomes, i.e., the magnitude of physiologic and clinical improvements. Recent evidence indicates concurrent ET leads to less than optimal improvements in muscle strength [17]. In addition, other studies have found concurrent ET leads to less than optimal improvements in peak oxygen uptake (VO_2_) in healthy males [18].

To our knowledge, it is unclear whether concurrent training can enhance or impair parameters of strength and aerobic capacity in patients with T2D. In addition, it is unclear if a concurrent ET training program implemented for a short period could produce positive adaptations, while considering aspects such as an individualized training approach during supervised sessions, taking into account the order of application. As such, addressing these issues is the primary objective of the current investigation. We hypothesize that a well-designed, short duration, concurrent ET program can lead to a reduction in HbA1c as well as improvements in peak VO_2_, peripheral muscle strength, and cardiovascular risk profile in a T2D cohort.

## Methods

### Design, Recruitment Process, and Population

Participants were recruited through word of mouth, radio advertisements, local e-mail subscription services, social media, and flyers. All potential participants were screened for exclusion criteria via telephone scripts and face-to-face interviews.

The current investigation recruited and enrolled a total of 41 patients (15 females and 19 males) with a confirmed diagnosis of T2D, aged 51 ± 7 years. Duration of T2D was based on a self-reported date of diagnosis.

The experimental procedures were performed in the Cardiopulmonary Physiotherapy Laboratory at the Federal University of São Carlos, and blood collection was performed at the Clinical Analysis Laboratory in the same location.

Eligibility criteria were (1) established diagnosis of T2D confirmed by a physician and (2) a sedentary lifestyle for at least 6 months according to the *American Heart Association* criteria [19]. Exclusion criteria consisted of (1) a confirmed diagnosis of any heart disease, (2) uncontrolled hypertension, (3) any musculoskeletal disorders, (4) any confirmed diagnosis of respiratory disease, and (5) confirmed autonomic neuropathy. Autonomic neuropathy was considered when the patient presented with a clinical diagnosis and/or heart rate >100 bpm [20] and a validated symptomatology 7 question tool [21]. The study followed the Declaration of Helsinki guidelines and was approved by the Human Research Ethics Committee of our Institution (protocol number 1318/1). The procedure was explained and subjects subsequently confirmed their participation in the study by written informed consent. Written informed consent was obtained from the patient for publication of this original manuscript and any accompanying images.

### Randomization

The current study is a randomized controlled trial (RCT) (RBR 492q8z) available from http://www.ensaiosclinicos.gov.br/rg/?q=RBR-492q8z. Allocation was performed by a person not involved in the study selecting the envelope from a box. Participants included in the study were randomized by 41 sealed opaque envelopes, 21 to the concurrent ET group (CART-G) and 20 to the sedentary group (SG). Subjects were randomized in an equal ratio (1:1) with a random block size of 4.

The data were analyzed by a specialist who was blinded to participant group allocation.

### Outcomes

Primary outcome: The primary outcome measures were change in HbA1c, peal VO_2_, and biochemical analyses in T2D patients after a 12-week concurrent ET program.

Secondary outcome: As a secondary outcome measure, we analyzed changes in peripheral muscle strength and body composition following a 12-week concurrent ET program.

### Procedures and Data Collection

All subjects were evaluated during the same time of the day (in order to consider differences in response due to circadian rhythm) in an experimental room with a temperature of 22 °C and relative air humidity between 50 and 60 %.

### Skinfold Thickness

Skinfolds of the biceps, triceps, subscapular, suprailiac, abdomen, and thigh were measured thrice using metal calipers (Cescorf, Porto Alegre, Rio Grande do Sul, Brazil). The average of the three skinfold measurements was used for calculation of percent body fat [22].

### Body Circumferences

The circumference of the arm, axillary, xiphoid, hip, waist, and thigh was measured using a flexible tape measure with 0.1-cm increments. Waist circumference was measured at the level of the umbilicus and the hips at the level of the iliac crest taken with the patient in a standing position. All measurements were performed thrice by a researcher who had been previously trained and certified to perform these procedures; the average of the three measurements was used for calculation [22].

Body composition was assessed by bioelectrical impedance analysis using the Tanita Bc533 and previously described methodology [23, 24].

### Biochemical Measurements

Blood specimens were obtained after an overnight fast for all measurements. HbA1c was measured in a central laboratory by anion exchange high-performance liquid chromatography (Variant II, BioRad, Berkeley, CA), coupled with a fluorescence detector method certified by the National Glycohemoglobin Standardization Program [25]. Fasting plasma glucose was measured by an enzymatic method using an AU 680^®^ (Beckman Couter, Suarlée (NAMUR) Belgium), and fasting plasma insulin was measured by a chemiluminescent assay (UniCel® DxI 800, Pasadena, CA). Total cholesterol (total-C), low-density lipoprotein cholesterol (LDL-C), high-density lipoprotein cholesterol (HDL-C), and triglycerides were measured by an enzymatic method AU 680® (Beckman Couter, Suarlée (NAMUR) Belgium) [26].

### Cardiopulmonary Exercise Testing (CPX)

A symptom-limited incremental exercise test was performed on a cycle ergometer (Recumbent Corival of MedGraphics, MN, USA) with the collection of gas exchange and ventilatory variables using a calibrated computer-based exercise system (Metabolic analyzer System Greenhouse telemetry module for field studies Oxycon-Mobile, Jaeger, Hoechberg, Germany).

The day before CPX, the subjects were taken to the experimental room for familiarization with the procedures and equipment to be used. All subjects were instructed to (1) avoid caffeinated and alcoholic beverages or any other stimulants the night before and the day of data collection, (2) not to perform activities requiring moderate-to-heavy physical exertion on the day before data collection, and (3) not to speak unnecessarily during the evaluation to avoid HR signal interference.

The exercise test consisted of (1) 5 min of rest, (2) 4 min with real “zero” workload, obtained by means of a system which moves the ergometer freewheel at 60 revolutions per minute (rpm), (3) the incremental phase, (4) 1 min active recovery period, and (5) 5 min passive recovery period. The workload (W) was continuously increased in a linear “ramp” pattern of 15 W min^−1^ [27], so that the incremental exercise testing duration was between 8 and 12 min [28]. The test finished when subjects reached physical exhaustion or demonstrated abnormal test responses that warranted test termination [27].

Peak VO_2_ was defined as the highest 15-s average value during the test [29]. Fifteen-second averaged ventilation (V_E_) and carbon dioxide production (VCO_2_) data, obtained from the initiation of exercise to peak, were inputted into a spreadsheet software (Microsoft Excel, Microsoft Corp., Bellevue, WA, USA). V_E_ and VCO_2_ responses throughout exercise were used to calculate the V_E_/VCO_2_ slope via least squares linear regression (*y* = mx + b, *m* = slope) [30].

### Isokinetic Strength Test

Isokinetic concentric knee-extensor strength on the dominant side was performed at a speed of 60°/s using an isokinetic dynamometer (Biodex Medical Systems 3, Shirley, NY). Positioning and stabilization of the subjects was standardized. Mechanical axis of rotation of the level arm was aligned to the axis of rotation of the joint being tested. The resistance pad at the end of the level arm was strapped to the most distal part of the tibia. Correction for the effect of gravity was made. After stretching, a warm-up exercise was performed on the dynamometer. The order of peripheral muscle tests applied was as follows: (1) Protocol 1, which was for the analysis of the maximal isokinetic strength, the volunteers performed five maximal efforts to determine maximal peak torque (in N m) at low angular velocity (U) of 60°/s; (2) Protocol 2, which was for the analysis of isokinetic endurance, the participants performed efforts for 1 min at a high angular velocity of 300°/s to determine average power (in W). This protocol assessed muscular efficiency and total work (in J) [31].

### One-Repetition Maximum Test (1-RM)

To determine protocol loads, the 1-RM test was applied by gradually increasing resistance until the volunteer succeeded in performing no more than one repetition [32]. The 1-RM test was performed for all eight exercises.

### Concurrent Aerobic and Resistance Training Program (CART)

The training protocol for the CART-G consisted of individually supervised sessions on alternate days, ~210 min/week, three times per week, for 12 weeks, totaling 36 sessions.

All training sessions were carried out under the individual supervision of exercise specialists. Each session lasted ~1 h and 10 min and included a (1) 5-min warm-up, (2) 60-min CART training, and (3) 5-min cool down. The protocol was performed for three separate sessions before determination of the 1-RM to allow familiarization of equipment. In order to avoid a bias effect during the training program, during each exercise session day, the subjects alternated the order of training (i.e., aerobic training first followed by resistance training and subsequently resistance training followed by aerobic training). Resistance training was performed at 60–80 % of predicted 1-RM [33] and consisted of eight resistance exercises targeting all major muscle groups as recommended by the *American Diabetes Association* [34]. Subjects performed sets of four upper body exercises (biceps free weights; seated row; triceps and, flat bench press) and three sets of four lower body exercises (leg press; leg extension; calf and, seated leg curl). Each maneuver consisted of three sets of 10–12 repetitions. The amount of weight lifted was progressively increased through sessions in order to maintain consistent repetitions during the training program. The participants were advised to rest 2 to 3 min between sets to allow for maximal muscle recovery.

Aerobic training was performed at the HR corresponding at 60–70 % of peak VO_2_ assessed by CPX. Each training session was 30 min in duration [34].

The workload during both aerobic and resistance training was gradually increased after experimental measures. Skinfold thickness, body circumferences, biochemical measurements, CPX isokinetic strength test, and the 1-RM test were assessed at the midway point of the protocol (i.e., sixth week). Table 1 lists the CART progression.

**Table 1:** Pearson correlation between HbA1c post X skinfold post in the CART group

	Aerobic		Resistance	
Weeks	Intensity (VO_2_) (%)	Duration (min)	Intensity	Sets/repetitions
1	60	30	60 % 1-RM	3/10
2	60	30	60 % 1-RM	3/10
3	65	30	70 % 1-RM	3/10–12
4	65	30	70 % 1-RM	3/10–12
5	70	30	75 % 1-RM	3/10–12
6	70	30	75 % 1-RM	3/12
7	70	30	75 % 1-RM	3/12
8	70	30	75 % 1-RM	3/12
9	80	30	80 % 1-RM	3/10
10	80	30	80 % 1-RM	3/10–12
11	80	30	80 % 1-RM	3/10–12
12	80	30	80 % 1-RM	3/12

Heart rate, blood pressure, and blood glucose measurements were monitored pre and post exercise sessions, in order to identify any adverse events. If blood glucose was >13.9 mmol/l (250 mg/dl) prior to exercise, the session was canceled. If blood glucose was >13.9 mmol/l more than two times in a same week, the subject was referred for a physician visit. All the subjects completed 36 sessions. Following the 12-week CART protocol, the subjects were re-evaluated.

All participants were instructed to maintain their usual medical care and they received dietary advice on healthy eating [35], but there was no specific dietary intervention with regard to eating habits. Subjects were given the opportunity to miss or cancel sessions with a makeup session scheduled as soon as possible. In this context, the duration of the program did not exceed 12 weeks.

### Statistical Analysis

Sample size calculation, based on a previous study [15], suggested that recruitment of 12 patients in each group would provide sufficient statistical power for the primary endpoint (i.e., peak VO_2_). The power (80 %) at an α-level of 0.05 to detect a significant difference in peak VO_2_ (ml kg^−1^ min^−1^) of ~1.2 ml kg^−1^ min^−1^ between groups was used.

The sample size was determined a priori through GPower software 3.1.9. Considering the main outcome was the mean difference between groups and times (analysis of variance (ANOVA): repeated measures, within-between interaction, effect size = 0.40, with *α* = 0.05, *β* = 80 %, *d* = 0.8) and the allocation ratio was 1:1, the calculated sample size was 16 for each group. Anticipating a dropout rate, more subjects were recruited in each group.

Data are presented as mean and standard deviation after testing for normal distribution by the Shapiro-Wilk test. Differences between the two groups (SG *versus* CART-G data) were assessed by unpaired Student’s *t* test.

Significance of between-group differences in medication changes was evaluated by Fisher’s exact test for categorical variables.

The effect of time (pre compared with post 3 months, p, i.e., effect of CART), group (SG compared with CART-G), and interaction between time and group effects were evaluated by two-way ANOVA for repeated measures.

Pearson’s correlations were tested to contrast the association between changes in variables pre and post training, followed by linear regression. All other data analysis was performed using the STATISTICA 5.5 software package (Stat Soft®) and SigmaPlot® 11. The level of significance was set at *P* ≤ 0.05 for all statistical tests.

The magnitude of the correlations was determined considering the following classification scheme for *r* values: ≤0.35 low or weak, 0.36 ≤ 0.67 moderate, ≥0.68 strong or high, ≥0.9 very high, and 1 perfect [36]. The probability of a type I error was established at 5 % for all tests (*α* = 0.05).

Subjects included in the final analysis had at least 80 % adherence to their exercise prescription over the 12-week protocol; there was 100 % adherence in CART-G

## Results

Sixty participants were screened; before the randomization, 12 more were excluded, 4 not meeting inclusion criteria, 4 declining to participate, and 4 living too far of a distance for data collection. Forty-eight subjects underwent randomization. Post-randomization but prior to training, two additional participants were excluded in the CART-G, one due to gynecologic surgery and the other voluntarily withdrew during the first week of training. Five withdrew from the SG post-randomization, one for personal reasons, one started to use insulin, and three moved to another city. All remaining 41 volunteers successfully completed the study, 20 in the control and 21 in the CART-G. No adverse events were identified during the protocol application. The randomization flow chart is illustrated in Fig. 1.

**Fig. 1:**
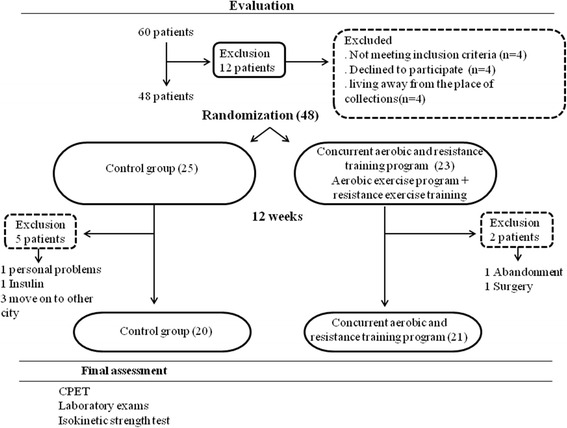
Flowchart with randomization study

As summarized in Table 2, there were no statistically significant differences in anthropometric and demographic characteristics, risk factors and/or medications between the two groups at baseline, demonstrating successful randomization.

**Table 2:** Anthropometric and demographic data, risk factors, and medications of the sedentary (SG) and the concurrent aerobic and resistance training groups (CART)

Variables	SG (*N* = 20)	CART-G (*N* = 21)	*P*
Age (years)	52.1 ± 7.3	49.5 ± 6.1	0.21
Height (cm)	167 ± 0.1	169 ± 0.1	0.53
Weight (kg)	85 ± 18.6	82.2 ± 19.4	0.63
BMI (kg/m^2^)	30.4 ± 5.8	30.1 ± 5.4	0.28
Duration type 2 diabetes (years)	7.4 ± 5.8	5.7 ± 5.5	0.17
SBP rest (mmHg)	131.1 ± 16.5	129.7 ± 15.8	0.79
DBP rest (mmHg)	89.4 ± 8.0	92 ± 11.1	0.40
HR (bpm)	83 ± 12.5	85.5 ± 13.1	0.54
Risk factors % (*n*)			
Tabagism	0	0	–
CAD family history	20 (4)	23.8 (5)	1.00
Dyslipidemia	50 (10)	42.8 (9)	0.75
Obesity	55 (11)	38 (8)	0.35
SAH	60 (12)	47.6 (10)	0.53
Medications			
Hypoglycemiants % (*n*)			
Biguanides	95 (19)	85.7 (18)	0.60
Sulfonylurea	45 (9)	47.6 (10)	1.00
Antihypertensive			
ACE I inhibitors	15 (3)	14.3 (3)	1.00
Renin inhibitors			1.00
ARAII	25(5)	20 (4)	0.71
Diuretics	5 (1)	9.5 (2)	0.60
Hypolipemiant			
Estatins	15 (3)	19 (4)	1.00
Fibrates	10 (2)	–	0.23

Data are reported as means ± SD. *BMI* body mass index, *SBP* systolic blood pressure, *DBP* diastolic blood pressure, *HR* heart rate, *CAD* cardiovascular disease, *SAH* systemic arterial hypertension, *ACE I* angiotensin converting enzyme I inhibitor, *ARAII* angiotensin II receptor antagonists

A two-way ANOVA was conducted to examine the effect of time (effect of pre and post 3 months) and group (SG vs. CART-G) on metabolic profile, glycemic control, peripheral muscular strength, and exercise capacity status. In Table 3, in relation to body composition, there were no differences in weight and BMI post exercise. However, visceral fat decreased significantly in the CART-G only (10.9 ± 4.1 to 9.7 ± 4.2–11 %).

**Table 3:** Summary of results pre and post 3 months

	SG (*N* = 20)		CARTG (*N* = 21)		Time factor	Exercise factor	Interaction
Variables	Pre	Post	Pre	Post			
Weight (kg)	85.0 ± 18.6	84.1 ± 17.9	82.2 ± 19.4	81.5 ± 19.5	0.64	0.06	0.80
BMI (kg/m^2^)	30.4 ± 5.8	30.1 ± 5.4	28.6 ± 5.2	28.3 ± 5.3	0.29	0.06	0.80
VF (%)	12.5 ± 5	12.7 ± 4.8	10.9 ± 4.1	9.7 ± 4.2‡†	0.10	0.16	0.04
HbA1c (mmol/mol )	71.7 ± 18.9	72.5 ± 20.9	65.4 ± 17.9	55.9 ± 12.7‡†	0.04	0.08	0.03
HOMA-IR	5.6 ± 4.7	5.2 ± 4.4	6.4 ± 6.8	5.0 ± 4.4	0.84	0.05	0.31
Cholesterol (mg/dl)	196.3 ± 44.2	216.9 ± 47.9*	198.3 ± 50.3	186.8 ± 35.1	0.33	0.35	0.002
LDL/HDL (mg/dl)	2.6 ± 0.9	3.0 ± 1.1*	2.5 ± 0.5	2.4 ± 0.6†	0.2	0.2	0.03
SBP rest (mmHg)	127 ± 16.9	131.7 ± 24.6	129.7 ± 16.3	118 ± 12‡†	0.24	0.34	0.02
DBP rest (mmHg)	89.3 ± 7.3	859 ± 13.8	92.1 ± 10.5	81.8 ± 6	0.82	<0.001	0.06
Skinfold thickness (cm)							
Triciptal	2.3 ± 1	2.1 ± 0.7	2.2 ± 0.6	1.6 ± 0.5‡†	0.16	<0.001	<0.001
Biciptal	1.4 ± 0.8	1.3 ± 0.6	1.3 ± 0.4	0.7 ± 0.3‡†	0.06	<0.001	<0.001
Subscapular	3.4 ± 1.1	3.6 ± 0.9	3.2 ± 0.9	2.4 ± 0.9‡†	0.02	<0.001	<0.001
Suprailiac	3.2 ± 1.3	3.5 ± 1.5	3.3 ± 1.1	2.2 ± 0.9‡†	0.11	0.003	<0.001
Abdominal	4.8 ± 1.8	4.7 ± 1.8	4.3 ± 1.6	3.1 ± 1.1‡†	<0.001	<0.001	<0.001
Thigh	2.8 ± 1.0	2.7 ± 0.9	3.0 ± 1.4	1.7 ± 0.8‡†	0.34	<0.001	<0.001
Cirtometry (cm)							
Arm	34.4 ± 4.2	36.1 ± 7.8	33.8 ± 3.7	33.3 ± 3.8	0.25	0.44	0.13
Axillary	108 ± 11.5	106.7 ± 11.6	105.9 ± 11.3	101.2 ± 13†	0.30	0.002	0.06
Xiphoid	102.2 ± 12.6	101.6 ± 12.5	99 ± 12.3	96.7 ± 12.5‡†	0.31	<0.001	0.02
Waist	100.8 ± 13.5	100.4 ± 13.3	95.3 ± 13	91.5 ± 13.6‡†	0.09	<0.001	<0.001
Umbilicus	105.2 ± 12.5	104.6 ± 12.1	98 ± 16.4	95 ± 11.9	0.04	0.15	0.32
Hip	104.2 ± 11.2	103.3 ± 8.6	102.9 ± 9.8	99 ± 8.7	0.33	0.02	0.17
Thigh	57.4 ± 5.4	56.7 ± 5.7	55.8 ± 7.5	56.7 ± 6.1	0.70	0.91	0.22

Data are reported as means ± SD. *BMI* body mass index, *HOMA-IR* homeostatic model assessment insulin resistance, *LDL/HDL* low-density lipoproteins/high-density lipoproteins, *SBP* systolic blood pressure, *DBP* diastolic blood pressure, *mmHg* millimeter of mercury. *P* ≤ 0.05, *sedentary group pre × sedentary group post; ‡exercise group pre × exercise group post, †sedentary group post × exercise group post

Also, HbA1c decreased by 9.9 % (65.4 ± 17.9 to 55.9 ± 12.7 mmol/mol) in the CART-G compared to baseline. Comparing the post-intervention difference between the SG and CART-G, the difference in HbA1c reached 17.1 % (72.5 ± 20.9 to 55.9 ± 12.7 mmol/mol). Moreover, LDL/HDL decreased in the CART-G (*P* < 0.05) while LDL/HDL and total cholesterol increased in the SG.

There was only a difference in resting systolic blood pressure (SBP) (129.7 to 118 mmHg) after CART-G, with no change in diastolic blood pressure (DBP).

All skinfolds decreased post exercise according to time and group [(i.e., interaction time (effect of exercise) vs. group (SG vs. CART-G), (*p* < 0.05)]. However, arm, umbilicus, hip, and thigh circumferences did not decrease after 3 months of exercise.

After 3 months, CART-G increased relative and absolute peak VO_2_ by 18.7 % [22.9 ± 6.1 to 27.2 ± 4.7 ml kg^−1^ min^−1^(*p* = 0.002)] as well as peak workload (*p* ≤ 0.001), V_E_ (*p* ≤ 0.001), VCO_2_ (*p* = 0.004), and the VE/VCO_2_ slope (*p* = 0.006). These statistical differences, according to time and group [(i.e., interaction time (effect pre and post) vs. group (SG vs. CART-G)], describe the simultaneous positive influence of a supervised, individualized CART program on VO_2_ (Table 4).

**Table 4:** Cardiopulmonary exercise testing responses pre and post 3 months

Variables	SG (*N* = 20)		CART-G (*N* = 21)		Time factor	Exercise factor	Interaction
CPX (peak)	Pre	Post	Pre	Post			
Workload (watts)	123.6 ± 36.9	122.1 ± 32.9	124.6 ± 29.1	149.9 ± 29‡†	0.19	<0.001	<0.001
V_E_ (l min^−1^)	74.2 ± 21.2	62.8 ± 14.0	72.4 ± 15.2	80.6 ± 13.7‡†	0.12	0.53	<0.001
VO_2_ (ml kg^−1^ min^−1^)	21.7 ± 4.5	21 ± 3.3	22.9 ± 6.1	27.2 ± 4.7‡†	0.02	0.02	0.002
VO_2_ (ml min^−1^)	1935.7 ± 581.1	1796.9 ± 485.4	2074 ± 443.8	2323.3 ± 523.8‡†	0.38	0.05	0.004
VO_2_ (ml min^−1^) % pred	108.8 ± 21.3	102.3 ± 20.5	111.8 ± 24.6	125.5 ± 27‡†	0.28	0.09	0.004
VCO_2_ (ml min^−1^)	2240.2 ± 612.6	2068.5 ± 444.1	2269.1 ± 478.7	2542.8 ± 485.5‡†	0.14	0.48	0.004
V_E_/VCO_2_ slope	32.6 ± 3.6	30.1 ± 3.1	31.6 ± 3.6	32.4 ± 3.1	0.55	0.17	0.006
RER	1.3 ± 0.1	1.2 ± 0.1	1.2 ± 0.1	1.2 ± 0.1	0.40	0.03	0.72
HR (bpm)	147.6 ± 13.9	139.9 ± 19.3	146.7 ± 12.9	149.8 ± 14.4	0.33	0.34	0.03
HR (bpm) % pred	91 ± 10	86 ± 15	84 ± 22	90 ± 11	0.36	0.95	0.03
O_2_ pulse (ml beat^−1^)	10.5 ± 3.6	10.9 ± 3.4	11.7 ± 2.1	12.8 ± 3.1	0.08	0.12	0.07
Dyspnea (0–10)	5.1 ± 2.9	4.3 ± 2.1	5.4 ± 2.4	6.1 ± 1.6	0.07	0.86	0.09
Leg effort (0–10)	4.7 ± 2.9	4.4 ± 2.2	5.1 ± 2.6	5.8 ± 2.6	0.19	0.70	0.32

Data are reported as means ± SD. *P* ≤ 0.05, sedentary group pre × sedentary group post; ‡exercise group pre × exercise group post, †sedentary group post × exercise group post

Finally, the CART-G demonstrated a significant improvement in peripheral muscle strength; peak torque to body weight ratio extensors by 17.2 % (167 ± 41.5 to 195.8 ± 43.6 %), peak torque to body weight ratio flexors by 32 % (87.9 ± 14.7 to 116 ± 30.1 %), and total work flexion increase by 26 % (1340.3 ± 453.8 to 1685 ± 717.1 J) (Table 5). Interestingly, the difference between groups in muscular strength, after 3 months, was observed only in the flexor muscles, also evidenced by peak torque to body weight ratio (SG = 89.5 ± 19.1 % vs. CART-G = 116 ± 30.1 %). The 1-RM test increased for all equipment when compared pre and post CART-G (Table 6).

**Table 5:** Isokinetic strength and endurance test responses pre and post 3 months

Variables	SG (*N* = 20)		CARTG (*N* = 21)		Time factor	Exercise factor	Interaction
Isokinetic strength test	Pre	Post	Pre	Post			
Peak torque extension (N m)	145.3 ± 47.9	143 ± 42.2	135.5 ± 41	159.7 ± 47.7‡	0.82	0.002	<0.001
Peak torque flexion (N m)	74.8 ± 26.4	72.2 ± 24.3	72.1 ± 19.3	92 ± 24.1‡†	0.27	<0.001	<0.001
PT/BW extension (%)	174.6 ± 34.3	176.4 ± 23.3	167 ± 41.5	195.8 ± 43.6‡	0.63	0.002	0.004
PT/BW flexion (%)	92.4 ± 19.8	89.5 ± 19.1	87.9 ± 14.7	116 ± 30.1‡†	0.10	0.001	<0.001
AVG power extension (watts)	90 ± 28	92 ± 27.3	86.8 ± 27	110.6 ± 31.8‡	0.42	<0.001	<0.001
AVG power flexion (watts)	50.2 ± 21.2	50.3 ± 19.5	47.7 ± 14.2	66.5 ± 18.2‡†	0.25	<0.001	<0.001
Isokinetic endurance test							
Total work extension (J)	2673.8 ± 960.3	2660.5 ± 895.2	2744 ± 783.8	3171.2 ± 1111.5	0.37	0.09	0.08
Total work flexion (J)	1325.2 ± 511.9	1343.2 ± 443.5	1340.3 ± 453.8	1685 ± 717.1‡†	0.33	0.03	0.05

Data are reported as means ± SD. *PT/BW* peak torque/body weight, *AVG* average force along the vertical axis. *P* ≤ 0.05, ‡exercise group pre × exercise group post, †sedentary group post × exercise group post

**Table 6:** One-repetition maximum responses pre and post CART

1-RM test	Pre (*N* = 21)	Post (*N* = 21)	*P* value
Lower body exercises			
Leg press	127.6 ± 21.9	174.3 ± 34.9	0.0001
Leg extension (right)	41.3 ± 14.2	59.3 ± 15.9	0.003
Leg extension (left)	40.1 ± 12.6	58 ± 16	0.002
Calf	91.6 ± 21.8	131 ± 27.5	0.0002
Seated leg curl (right)	31.6 ± 11.2	43 ± 11.5	0.01
Seated leg curl (left)	31.9 ± 10.8	42 ± 9.5	0.021
Upper body exercises			
Biceps	10.6 ± 2.2	13.7 ± 2.9	0.005
Flat bench press	39 ± 13	52.7 ± 15.1	0.017
Triceps	97.4 ± 20.7	120.8	0.001
Seated row	62.5 ± 18.2	84.3 ± 21.5	0.010

Data are reported as means ± SD. *P* ≤ 0.05

Peak VO_2_ was correlated negatively with HbA1c (Fig. 2). Correlations between triceps, biceps, subscapular, suprailiac and, abdominal skinfolds, and HbA1c were low to moderate (Table 7). No significant correlations were found between parameters of body composition, strength and insulin resistance.

**Fig. 2:**
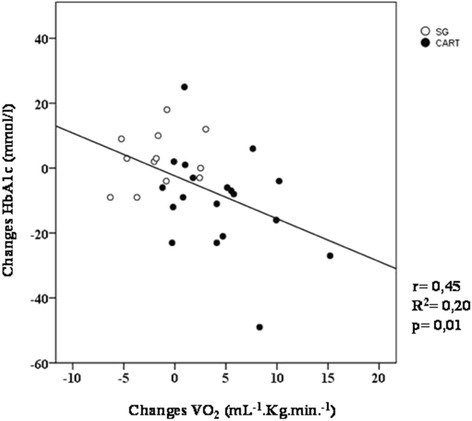
Comparison between changes of VO_2_ (ml kg^−1^ min^−1^) and changes HbA1c (mmol/mol) in response to 12 weeks of CART (*filled circles*) and sedentary (*open circles*) in diabetic patients

**Table 7:** Pearson correlation between HbA1c post X skinfold post

Skinfolds (cm)	HbA1c	
	*P*	*r*
Triciptal	0.04	0.32
Biciptal	0.04	0.33
Subscapular	0.0007	0.53
Suprailiac	0.01	0.41
Abdominal	0.01	0.39

Data are reported as mean ± SD. *P* ≤ 0.05

## Discussion

Several aspects making this an innovative investigation (1) demonstrated that a 12-week supervised, individualized, and personalized CART significantly improved (1) glycemic control, (2) body composition, (3) peak VO_2_, and (4) muscular strength. Additionally, peak VO_2_ was correlated with HbA1c. This is the first study, to our knowledge, that assessed CART using an individualized, supervised approach over a 12-week period. Furthermore, there was 100 % adherence to the program.

In type-1 diabetes patients with poor glycemic control, Tagougui et al. concluded that the glycation of hemoglobin itself could play a role in reducing the gain in peak VO_2_. Additionally, it was demonstrated that poor glycemic control during exercise blunted the deoxyhaemoglobin (HHb) increase, indicating lower muscle oxygen extraction and a higher oxygen affinity for glycated hemoglobin [37]. Furthermore, it has been shown that increased VO_2_ peak is associated with decreased cardiovascular and all-cause mortality [38]. From a clinical perspective, our data indicate that CART can be an effective strategy in reducing cardiovascular risk factors with potential beneficial effects on VO_2_ and peripheral muscle strength in patients with T2D.

### Effect of CART on Body Composition

The findings of the current study demonstrated that body weight and BMI remained stable in both groups. This is consistent with several previous studies [39–41], demonstrating no difference in the body habitus characteristics post ET with a longer training duration (9–12 months) than the present study. However, Church et al. studied the benefits of aerobic training, resistance training, and a combination of both on HbA1c in individuals with T2D and found that the combined training group had a decrease in mean weight compared to the control and resistance training group [15]. Recently, however, Johannsen et al. found that a resistance training program for 9 months decreased body weight more than aerobic exercise; the authors attributed this finding to the increase in free fatty acids [42]. From the literature in comparison to the current study, we observed that previously reported training duration assessed was typically longer although a significant change in body weight was not common.

Body weight collectively includes not only body fat but also muscle, bone, and water. From this perspective, skinfold thickness measures may be a more sensitive and direct measure of cardiovascular risk profile (i.e., more accurately identifying adiposity profile). Moreover, changes in adiposity or total body fat are more relevant regarding risk reduction compared to quantification of body weight reduction alone [43]. In the present study, despite the fact that body weight did not change, we observed that all skinfold measurements decreased and muscular strength improved, with these data collectively speculating that CART might reduce adiposity and increase lean body mass, which are considered to be highly favorable adaptations in patients with T2D.

In this way, Boule et al. performed a meta-analysis that systematically reviewed and quantified the effect of exercise on HbA1c and body mass in T2D, concluding that exercise programs longer than 8 weeks reduce HbA1c, although there appears to be no change in body mass [44].

### Effect of CART on Glycemic Control

Surprisingly, at baseline, all study subjects had a poor glycemic control, as demonstrated by HbA1c, which remains the gold standard for assessing glucose homeostasis as an integration of both fasting and postprandial glucose variations over a 3-month period [45]. Although all subjects were diagnosed with T2D, they are under use of hypoglycemiants, some using more than one. Thus, we expected that this cohort presented with better glycemic control. In addition, all the participants were under the routine care of an endocrinologist and seen on a monthly basis for medical management.

Although few studies has found an improvement in HbA1c after a combined ET program, the subjects enrolled were prescribed insulin and utilized pneumatic machines [46] or elastic bands [47] or the time of the intervention was longer [14, 15]. Thus, the protocol used in the current study, and therefore the results, may not be generalizable to these previous protocols. In addition, Church et al. and Sigal et al. found that a long period of combined ET program could beneficially modulate HbA1c. However, our study seems to be the first to find an important reduction in HbA1c using short-term CART, which has implications for program costs needed for implementation [14, 15].

Each 1 % increment of HbA1c corresponds to a 29 mg/dl increase in mean glucose concentration [48]. Conversely, an absolute decrease of 1 % in HbA1c levels has been associated with a 15 to 20 % decrease in cardiovascular events [49]. To our knowledge, this current study is the first randomized trial to demonstrate as profound a decrease in HbA1c (9.9 %) when compared to baseline using a CART protocol and a 17.1 % when comparing the CART-G to the SG following the trial.

More in line with the present study, Church et al. after 9 months of aerobic and resistance training, when compared to a non-exercise control group, showed a reduction in HbA1c levels [15]. Sigal et al. demonstrated the same benefits, but after 22 weeks, where HbA1c decreased by approximately 13 %. In the present study, considering the specificity of our CART program, we demonstrated the same benefits in a shorter time frame [14]. The established HbA1c goal by an International Expert Committee [50] is 6.5 % or 47.5 mmol/mol. As such, CART may prove to be a valuable intervention strategy to achieve this goal. Even so, in many patients with T2D, with all the efforts and intensive treatment, optimal glycemic control may be difficult to achieve [51, 52].

### Effect of CART on Cardiorespiratory and Metabolic Profile

Awotidebe et al. previously demonstrated that patients with T2D demonstrated a lower peak VO_2_ compared with healthy controls [53]. Our findings show that exercise training was associated with significant increase in VO_2_ peak, which was also correlated to the decrease in HbA1c. These findings collectively indicate that in addition to a significant improvement in aerobic capacity with ET [54], there is increased insulin action [55] and improved blood glucose levels [14], which could be due to upregulation of GLUT-4 transporter expression at a cellular level. This mechanistic pathway is of great clinical importance, indicating the particular benefit of a concurrent ET program in patients with T2D.

Similar results were demonstrated by Larose et al. in the DARE trial, where T2D patients were randomly allocated into aerobic, resistance, or aerobic plus resistance training groups. This study demonstrated a link between positive changes in VO_2_ and HbA1c in the combined aerobic and resistance training group only [56]. The authors speculated this combined training program uniquely resulted in favorable adaptations in glucose metabolism at the cellular level [56].

In the present study, we observed that changes in peak VO_2_ could predict 21 % of the changes expected for HbA1c, which can explain the link between metabolic profile and exercise performance using the CART program. In parallel, Sénéchal et. al. reporting results from the HART-D study, demonstrated an association between the change in VO_2_ (quantified by estimated peak METs) and a reduction in central adiposity were the most prominent predictors of the change in HbA1c in response to a 9-month ET program [54].

### Effect of CART on Muscle and Endurance Strength

It is already known that the skeletal muscle is responsible for approximately 75 % of whole body insulin-stimulated glucose uptake [57]. In the current study, after CART, the subjects showed improved muscle strength, demonstrated by peak of torque to body weight ratio (increase of 17.2 % in extensor and 32 % in flexor) as well as improved endurance of leg flexors (26 %). These results, associated with a reduction in thigh skinfold measurements, we speculated may indicate an increase in muscle mass, although direct measurements of lean mass gains were not explored in this study. This finding also has a high clinical relevance, particularly considering a decreased muscle mass is considered to be a major impairment among patients with T2D [58]. Our findings are also consistent with Maiorana et al., investigating the effect of a short-term 8-week circuit training program composed by aerobic and resistance exercise with T2D in a randomized crossover protocol, which observed an increase of 12 % in muscle strength [59]. However, Church et al. did not observe a significant improvement in peak torque and work in the combined training program after 9 months.

Sousa et al., in a RCT evaluated the impact of different exercise training modalities on functional fitness responses in apparently healthy older men, showing that only the combined exercise program was effective in improving all functional fitness components. It is noteworthy to point out they used long-term training (9 months) yet did not evaluate muscle strength [60].

Untrained individuals normally have rapid improvements in muscle strength during the initial phases of a training program, which are predominantly attributed to a learning effect as well as neuromuscular adaptations within the first 4 weeks of program initiation [61]. In this context, our results can be explained by true adaptations in muscle force production since the patients continued improving muscle strength throughout the program. These aspects are important given a recent study contrasting different ET protocols (i.e., aerobic versus resistance) observed that only resistance exercise had an important influence on maintaining reductions in LDL-C, HbA1C and the increase in HDL-C, even if the training program is interrupted for a short term [62].

Resistance training have been highly recommended by the ACSM as an important tool to preserve/improve muscle mass and potentially improve insulin sensitivity, not only due to development of more muscle mass but also due to improved muscle quality [63, 64]. Combining aerobic with resistance training, alternating the training each session can be an important alternative to preserve gains of functional capacity and muscle strength status.

In fact, it has been shown that the supervised aerobic, resistance or combined aerobic/resistance training are associated with greater decreases in HbA1c compared to exercise counseling alone or a singular training approach. Umpierre et al., in a systematic review with meta-regression analysis of RCTs, found that the chronic impact on glycemic control might be affected by volume and intensity of the structured exercise program and not only the mode of exercise training [65].

The main drawback of our study was the impossibility to use a gold standard assessment to diagnose autonomic function. This would have been valuable given T2D has a negative impact on the autonomic control. Assessing the effects of CART on autonomic function would have been of value and should be performed in future investigations. Another potential limitation was the impossibility to have a gold standard body composition assessment, such as DEXA. Thus, the inferences related to these assessments in our study must be confirmed by future investigations.

## Conclusions

In conclusion, this study showed that a 12-week personalized and supervised concurrent ET program can improve not only the VO_2_ and muscle performance, but also glycemic control and body habitus. Future trials are necessary to contrast different approaches to CART, considering the effect on order of training program in the T2D population.
